# LDsplit: screening for *cis*-regulatory motifs stimulating meiotic recombination hotspots by analysis of DNA sequence polymorphisms

**DOI:** 10.1186/1471-2105-15-48

**Published:** 2014-02-17

**Authors:** Peng Yang, Min Wu, Jing Guo, Chee Keong Kwoh, Teresa M Przytycka, Jie Zheng

**Affiliations:** 1 Bioinformatics Research Centre (BIRC), School of Computer Engineering, Nanyang Technological University, 50 Nanyang Avenue, Singapore 639798, Singapore; 2 Institute for Infocomm Research (I2R), A*STAR (Agency for Science, Technology, and Research), 1 Fusionopolis, Singapore 138632, Singapore; 3 NCBI, NLM, National Institutes of Health, 8600 Rockville Pike, Bethesda, Maryland 20894, USA; 4 Genome Institute of Singapore, A*STAR, Biopolis, Singapore 138672, Singapore

**Keywords:** Meiotic recombination hotspots, Single nucleotide polymorphism (SNP), DNA sequence motif, Genome instability, Linkage disequilibrium (LD)

## Abstract

**Background:**

As a fundamental genomic element, meiotic recombination hotspot plays important roles in life sciences. Thus uncovering its regulatory mechanisms has broad impact on biomedical research. Despite the recent identification of the zinc finger protein PRDM9 and its 13-mer binding motif as major regulators for meiotic recombination hotspots, other regulators remain to be discovered. Existing methods for finding DNA sequence motifs of recombination hotspots often rely on the enrichment of co-localizations between hotspots and short DNA patterns, which ignore the cross-individual variation of recombination rates and sequence polymorphisms in the population. Our objective in this paper is to capture signals encoded in genetic variations for the discovery of recombination-associated DNA motifs.

**Results:**

Recently, an algorithm called “LDsplit” has been designed to detect the association between single nucleotide polymorphisms (SNPs) and proximal meiotic recombination hotspots. The association is measured by the difference of population recombination rates at a hotspot between two alleles of a candidate SNP. Here we present an open source software tool of LDsplit, with integrative data visualization for recombination hotspots and their proximal SNPs. Applying LDsplit on SNPs inside an established 7-mer motif bound by PRDM9 we observed that SNP alleles preserving the original motif tend to have higher recombination rates than the opposite alleles that disrupt the motif. Running on SNP windows around hotspots each containing an occurrence of the 7-mer motif, LDsplit is able to guide the established motif finding algorithm of MEME to recover the 7-mer motif. In contrast, without LDsplit the 7-mer motif could not be identified.

**Conclusions:**

LDsplit is a software tool for the discovery of *cis*-regulatory DNA sequence motifs stimulating meiotic recombination hotspots by screening and narrowing down to hotspot associated SNPs. It is the first computational method that utilizes the genetic variation of recombination hotspots among individuals, opening a new avenue for motif finding. Tested on an established motif and simulated datasets, LDsplit shows promise to discover novel DNA motifs for meiotic recombination hotspots.

## Background

During meiosis, homologous recombination is initiated by SPO11-catalyzed machinery at DNA double strand breaks. In many species, recombination events are clustered in localized regions of a few kb long, called recombination hotspots. Understanding the regulatory mechanisms of recombination hotspots can shed light on birth defect diseases, molecular evolution, genome instability, *etc*. [1,2]. Therefore, it is desirable to understand how the locations and intensities of recombination hotspots are regulated.

For the regulatory mechanisms of recombination hotspots, striking progress has been made recently, thanks to the high-throughput genomic technology and bioinformatics techniques. Myers *et al.* used the LDhat software to estimate the fine-scale genome-wide recombination hotspots from HapMap Phase II SNP data [3]. From the hotspots, a list of motifs has been discovered, in which the two most prominent motifs are the 7-mer CCTCCCT and the 9-mer CCCCACCCC. Interestingly, when located inside THE1A/B repeats, the motifs have much stronger association with proximal hotspots than outside the repeats. The 7-mer motif was later extended to a degenerate 13-mer motif CCNCCNTNNCCNC [4]. Moreover, the 13-mer motif contains the sequence pattern of 3-periodicity, indicating a zinc finger binding array. Then, three groups reported the discovery of PRDM9 protein as a *trans*-acting regulator of the locations and intensities of meiotic recombination hotspots in human and mouse [5-7]. Strikingly, PRDM9 protein binds to the aforementioned 13-mer motif. The discovery of PRDM9 protein and its binding motifs was a major breakthrough in the understanding of the regulation of meiotic recombination hotspots. However, it has been observed that PRDM9 is not indispensable for recombination hotspots; for example, in PRDM9 knockout mouse germ line, meiotic recombination hotspots are still observed [8,9]. Overall, PRDM9 can explain about 18% of the variations in human recombination hotspots [6] and the 13-mer motif occurs in about 40% of human hotspots [4]. Therefore, additional *trans* and *cis*-regulatory elements for meiotic recombination hotspots remain to be discovered. Some recent efforts have been made toward this goal [10-12]. Moreover, follow-up investigation of the functions of PRDM9, *e.g.* its detailed mechanism to mediate the location and intensity of meiotic recombination, is also under intense research [9,13,14].

The discovery of DNA sequence motifs that stimulate meiotic recombination is crucial to the uncovering of the regulatory mechanisms of recombination hotspots. Several approaches have been developed for this purpose. The first approach is based on yeast mutagenesis. After genetically mapping the locations of meiotic recombination hotspots, Steiner *et al.* carried out base-pair substitution screening on the genome of fission yeast to scan for DNA sequences responsible for the activities of hotspots [15]. They identified 5 DNA motifs stimulating recombination hotspots, and showed evidence for the existence of more motifs. The second approach is the computational search for short DNA sequences that are significantly enriched in hotspots against cold spots [3,4]. Its success in identifying the 13-mer binding motif of the PRDM9 protein shows the power of bioinformatics methods for the study of genetic recombination [5]. However, this approach has several caveats. First, the statistical associations based on counting of co-localization of hotspot and motif may not correspond to biological causal relations. Second, due to the limited power of computational detection of hotspots based on LD patterns, and the difficulty of finding degenerate motifs genome-wide, false negative remains a serious issue. The discovery of the 13-mer motif was based on two exact shorter motifs (CCTCCCT and CCAC) with two bases in between, which required close manual inspections [4].

It is desirable and challenging to design Bioinformatics algorithms to automatically detect degenerate motifs for recombination hotspots in large-scale. To this end, additional information is needed to increase the statistical power of motif detection. One type of such information is the association between sequence polymorphisms with the variation in recombination rates of a hotspot. Evidence of such association has been demonstrated by sperm typing. For instance, individuals with different alleles at the FG11 SNP located within the DNA2 hotspot have 20-fold difference in recombination rate of the DNA2 hotspot [16]. A similar case was reported for the NID1 hotspot [17]. Myers *et al.* noted that the two SNPs associated with the DNA2 and NID1 hotspots discovered by sperm typing are located within the motifs (CCTCCCT and CCCCACCCC) after the motifs had been identified; however, information of sequence polymorphism did not contribute to the discovery of motifs. In extending the 7-mer motif to the degenerate 13-mer motif [4], Myers *et al.* did compare the original and disrupt forms of motifs due to mutations, however they only compared motif occurrences in *different* hotspots in the human reference genome, rather than polymorphisms of the *same* motif occurrence and variations of intensities at the *same* hotspot (*e.g.* DNA2 and NID1) among different individuals. Therefore, in the above computational approach, genetic variation information encoded in the DNA sequence polymorphisms has not been fully used for motif discovery.

Recently we have designed an algorithm called LDsplit to detect DNA sequence polymorphisms associated with individual meiotic recombination hotspots, and thereby discover *cis*-regulatory elements of these hotspots [18]. Given a sample of haplotypes (*e.g.* about 200 consecutive SNPs from HapMap Phase 2 data) flanking a hotspot *H*, LDsplit first splits the haplotypes into two subsets according to alleles of a candidate SNP *A* (*i.e.* haplotypes in each subset have the same allele at that SNP). Then, LDsplit compares the population recombination rate of the hotspot *H* between the two subsets calculated by LDhat algorithm [19]. The rationale is that, if the SNP *A* is located in a *cis*-regulatory element (say, a motif) of the hotspot *H*, then one allele of SNP *A* represents a mutation that disrupts the motif. As a result, the subset of haplotypes with that disruptive allele should have lower intensity of hotspot *H* than the other allele. This is the case for the sperm typing examples of DNA2 hotspot and NID1 hotspot. This method is based on the assumption that population genetics methods (*e.g.* LDhat) for estimation of recombination rate by inferring historical crossovers from LD patterns should be able to capture the extant difference of hotspot intensities between the two alleles, which has been supported by some evidence [3]. Extensive tests on simulated and real data demonstrated the effectiveness of LDsplit to capture *cis*-regulatory genomic loci associated with recombination hotspots [18]. However, the software implementation of LDsplit needs an accessible user interface to be shared with research community, and it needs further validation with real data.

In this paper we report a significant upgrading to the implementation of LDsplit algorithm, and also demonstrate that LDsplit can help discover DNA sequence motifs stimulating recombination by narrowing down to *cis*-regulatory genomic regions. First, we have implemented a user-friendly graphic interface (GUI) for the LDsplit algorithm. Compared with the previous implementation using Perl, this version is implemented in Java, which is more efficient and platform independent, running under Windows, Linux and Mac OS. It provides computational unsophisticated users from life sciences a convenient access to the software. More importantly, the integrative scientific visualization of genetic data (*e.g.* recombination rate profile, physical locations of SNPs, shape of recombination hotspots) shows informative details about the genomic context of hotspots, which greatly facilitate explorative analysis of genetic factors of recombination hotspots. Far more than being incremental, this upgrading significantly enhances the usefulness and impact of LDsplit algorithm for the biomedical research. Second, to demonstrate the effectiveness of LDsplit, we carry out a focused analysis of SNPs falling inside the well known 7-mer motif CCTCCCT, which is the core of the binding motif of PRDM9. Running LDsplit on HapMap (phase 2) SNPs flanking the 7-mer motif, we observe that the original form of the 7-mer motif tends to have higher LD-based recombination rates than the disrupt form (*i.e.* disrupted by the alternative allele of the SNP inside the 7-mer motif). This is evidence that, in spite of biased gene conversion, LDsplit is able to capture the allele-specific intensities of hotspots. Third, to validate LDsplit by demonstrating its ability to uncover the 7-mer motif, we picked haplotype windows of about 200 SNPs each flanking a hotspot containing an occurrence of the 7-mer motif CCTCCCT, and then ran LDsplit on each SNP window. From the output of LDsplit, SNPs with the most significant p-values are extended to short DNA sequences, which were fed into the motif-finding algorithm of MEME [20]. We found that the top two motifs found by MEME from the top SNPs given by LDsplit match closely the 7-mer motif. In contrast, when running on DNA sequences flanking SNPs randomly chosen from the windows (*i.e.* without guide of LDsplit), none of the output motifs is similar to the 7-mer motif. This test on the well known 7-mer motif shows that LDsplit can narrow down to target *cis*-regulatory elements for meiotic recombination, which significantly increases the power of motif-finding algorithms like MEME [20]. Moreover, we carried out simulation studies by inserting two artificially generated motifs near SNPs simulated to be associated with hotspots. LDsplit is able to guide MEME to identify both motifs as top hits. Therefore, LDsplit can be used to discover novel DNA sequence motifs, including hotspots of human and mouse that have not been covered by PRDM9 and many species whose *cis*-regulatory motifs for recombination hotspots are yet unknown.

## Implementation

**Figure 1:**
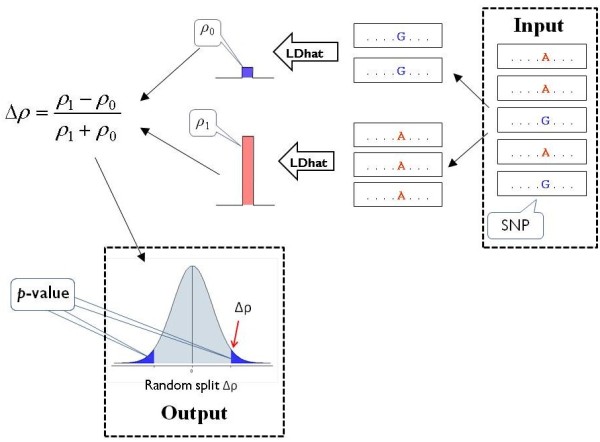
**Workflow chart of the LDsplit algorithm.** For each candidate SNP, LDsplit first divides the haplotype data into two subpopulations by SNP alleles. Then it calls LDhat to estimate the recombination rates for each subpopulation, and calculates the differences (Δ*ρ*) of hotspot strengths between the SNP alleles as (*ρ*_0_ − *ρ*_1_)/(*ρ*_0_ + *ρ*_1_), where *ρ*_0_ and *ρ*_1_ denote hotspot strengths in different subpopulations. Finally, the p-value of association is estimated by comparing the observed Δ*ρ* with the null distribution of random Δ*ρ* simulated by permutation tests.

The rationale of LDsplit is that, if a SNP is associated with a recombination hotspot, then between two alleles of the SNP the strengths of the hotspot are likely to be different. It is based on the assumption that historical recombination rate estimated by LD-based computational methods can approximate the extant recombination rate accurately. Based on this rationale, the LDsplit algorithm is designed as follows (Figure 1). For each candidate SNP, LDsplit first divides the population of chromosomes into two subpopulations by SNP alleles (*i.e.* all chromosomes in one subpopulation have the same allele). Then it calls the rhomap program (which is designed to estimate variable recombination rates in the presence of hotspots) in the LDhat package (see [19]) to estimate the recombination rates for each subpopulation, and calculates the normalized difference (Δ*ρ*) of hotspot strengths between the SNP alleles as (*ρ*_0_ − *ρ*_1_)/( *ρ*_0_ + *ρ*_1_), where *ρ*_0_ and *ρ*_1_ denote the strengths of the hotspot in two different subpopulations. The p-value of the hotspot-SNP association is estimated by comparing the observed Δ*ρ* with the null distribution of random Δ*ρ* simulated by permutation tests (*i.e.* each time it randomly splits the population into two pseudo-populations to calculate a random Δ*ρ* value).

The implementation of LDsplit consists of two stages. First, LDhat is called to calculate the recombination profiles for subpopulations split by SNP alleles and pseudo-population in permutation tests. Second, the p-values of hotspot-SNP associations are estimated and the recombination profiles of sub-populations are displayed. In the rest of this section, we will describe the two stages of LDsplit in detail. The source code and user manual of LDsplit are available for free download on its website (http://www.ntu.edu.sg/home/zhengjie/software/LDsplit.htm).

Stage 1: Calculating recombination profiles

We apply LDhat to calculate recombination profiles for a window consisting of sequences of SNPs (or haplotypes). There are three types of recombination profiles: (1) the profile of the whole input population of haplotypes; (2) profiles of sub-populations of haplotypes each corresponding to an allele of a candidate SNP (*i.e.* for each SNP, splitting the population into two sub-populations according to the two alleles of the SNP); (3) profiles of pseudo-populations from a random split of the input population. Meanwhile, the input SNP data in this stage consist of two text files, namely the sites file (consisting of haplotypes in FASTA format) and the locs file (consisting of physical locations of the SNPs on the chromosomes). Both types of files can be extracted from HapMap SNP database by cutting a window of haplotypes. LDsplit provides a friendly user interface to cut a window from HapMap data and generate the sites and locs files. In addition, the user can set the parameters involved in this stage (instructions provided in the user manual). As LDhat is computationally costly, this stage of LDsplit usually takes a long time on a regular personal computer (*e.g.* a few hours for 180 haplotypes each of 200 SNPs). Hence it is better to batch this computation. The output of this stage can be exported to hard disk as a Java serialization file, which can be loaded back into LDsplit for analysis in the next stage.

Stage 2: Deriving hotspot-SNP associations

**Figure 2:**
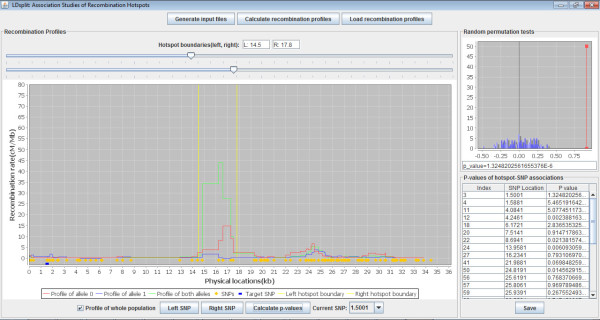
**Screen shot of the graphical user interface of LDsplit.** Please refer to the user manual of LDsplit for software usage, graph plot description and table with p-value. It can be downloaded from:http://www.ntu.edu.sg/home/zhengjie/software/LDsplit/LDsplit_manual.pdf.

Using the data output in the first stage, LDsplit visualizes the recombination profiles of the whole and sub-populations of chromosomes for exploratory analysis. To estimate hotspot-SNP associations, users first choose the boundaries of a hotspot using two sliders. For the chosen hotspot, LDsplit calculates the p-value of its association with every proximal SNP with minor allele frequency (MAF) no less than a threshold (*e.g.* 30%) in the window. For every candidate SNP, users can browse the recombination profiles of its two alleles (shown as blue and red lines in Figure 2). The physical positions of SNPs are shown as yellow dots below the recombination profiles, and user can click buttons (labelled “Left SNP” and “Right SNP”) to navigate among candidate SNPs (MAF ≥ 30%) and inspect their allele-specific recombination profiles. Moreover, the histogram of random Δ*ρ* values from the permutations tests and the observed Δ*ρ* value for a SNP chosen by users will be displayed in a window on the top-right panel, illustrating how p-values are calculated. A table on the bottom-right panel displays the p-values and positions of all candidate SNPs, and user can save it to a text file for further analysis (Figure 2). To identify DNA sequence motifs associated with recombination hotspots, one can search for DNA sequence patterns conserved in proximal regions of the identified SNPs using some motif-finding program such as MEME [20].

The software of LDsplit was implemented in Java, with a user-friendly graphical interface (as shown in Figure 2). For detailed instructions of using LDsplit, please see its user manual included in the package of LDsplit.

### Simulation

To demonstrate that LDsplit can help narrow down to hotspot associated motifs, we simulated recombination hotspots and flanking SNPs using simuPOP (version 1.0.3), a forward-time population genetics simulation framework [21]. In each test, a simuPoP based Python script was run to simulate the evolution of a population of around 5,000 individuals during many generations (for example, 3,000) using a forward-time model. Each individual had a pair of homologous haplotypes spanning 200 kb of DNA. Each haplotype was represented as a list of SNPs with alleles 0 and 1.

A simulated hotspot was located at the central point of haplotypes, and a causal SNP, whose alleles resulted in different probabilities of crossover at the recombination hotspot, was inserted at the position of 100 kb. In each generation, we randomly selected pairs of individuals as parents. Assuming one crossover event in a chromosome of 200 Mb per meiosis, in 200 kb the background probability of a crossover would be 0.001. When the hot allele of the causal SNP is present, the probability of a crossover would be increased to 0.01. In addition, the crossover position was chosen with the probability under normal distribution with mean at position 100 kb where the center of hotspot is located. At the end of simulation, a simulated population was exported, from which we randomly collected 10 subsets, each consisting of 90 individuals (180 haplotypes) as benchmark SNP data. The SNPs were extended with randomly generated DNA sequences, and artificial motifs were inserted spanning the causal SNPs. The SNP and DNA sequence data would then be fed into LDsplit and MEME to test if they are able to identify the motifs.

## Results and discussion

As a case study to demonstrate the usefulness of LDsplit software for the discovery of *cis*-regulatory motifs of meiotic recombination hotspots, we will analyze the 7-mer motif of CCTCCCT, which has been established as the core binding motif of PRDM9. First, running LDsplit, we confirm that, when the 7-mer motif containing a SNP is disrupted by one allele of the SNP, its proximal hotspot would have lower intensity estimated from LD patterns. Second, we show that LDsplit can guide the discovery of the 7-mer motif by narrowing down to genomic regions proximal to motifs. Third, we also discuss the effects of biased gene conversion implicated in this study. Then, LDsplit is further validated through simulation studies. At the end, we outline directions for future work.

### Disrupting effect of SNPs inside motifs

As introduced in the background section of this paper, the DNA binding of PRDM9 protein regulates the locations and intensities of many recombination hotspots of human and mouse. Particularly, the 7-mer DNA sequence motif CCTCCCT is at the core of the 13-mer binding motif of PRDM9 in the human genome [4]. Thus, this 7-mer motif has the function of stimulating human recombination hotspots, which will be confirmed by LDsplit in this section.

First, we search in the human genome for all occurrences of the 7-mer motif that each contains a SNP (using data from HapMap Phase 2 release 22 and human genome Build 36.1, hg18). We have not observed any case that one occurrence of the 7-mer motif contains more than one SNPs. In our previous study [18] we focused on the chromosome 6 of Asian (Chinese and Japanese) population from HapMap phase 2, and obtained successful results. Instead of repeating our past results, we chose SNPs in the European population instead and excluded chromosome 6 from the HapMap Phase 2 data. In this way, we collected 228 occurrences of the 7-mer motif each containing a SNP. Second, from the 228 SNPs we selected those SNPs with MAF (minor allele frequency) at least 30% in the European population, so that LDsplit will not give biased prediction due to small numbers of haplotypes of the minor allele. After this filtering, 70 SNPs inside the 7-mer motif were left, which are proximal to 15 recombination hotspots Additional file 1. Details of the 70 SNPs and their flanking motif occurrences can be find in Additional file 2 "70_SNP_motif_info.xlsx". Then, we extended each of the 70 selected SNPs to a flanking window of 101 SNPs (with 50 SNPs on each side), and ran LDsplit on haplotypes of the 101-SNP window. From the results of LDsplit on this window, we identified the hotspot *H* that is located nearest to the candidate SNP *A* (at the center of the window and falling inside the motif). In the following analysis, we will compare the recombination rates of hotspot *H* between two alleles of the SNP *A*, where one allele preserves the original form of the 7-mer motif, and the other allele disrupts the motif.

**Figure 3:**
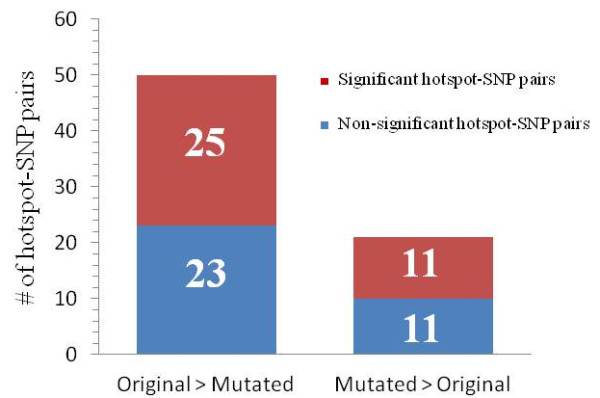
**Distributions of two types of Hotspot-SNP pairs where the SNPs are located inside the PRDM9 motif of CCTCCCT.** On the left side, for each hotspot-SNP pair, the SNP allele that preserves the motif (called “original”) corresponds to a higher recombination rate than the SNP allele that disrupts the motif (called “mutated”). The right side is the opposite. The red boxes contain the numbers of hotspot-SNP pairs with significant LDsplit p-value, and the blue boxes contain numbers of non-significant pairs.

Based on the estimated recombination rates from LDsplit, we can divide the above 70 SNPs into two categories: One is the *positive* set in which the SNP allele preserving the original form of the 7-mer motif corresponds to a higher estimated recombination rate than the other allele; and the rest SNPs are in the *negative* set, *i.e.* the disrupting allele has the higher recombination rate. As shown in Figure 3, the positive set contains 48 SNPs while the negative set contains 22 SNPs. The fact that the positive set is bigger than the negative set indicates that the disruptions to the 7-mer motif by SNPs indeed correspond to weakened intensities of recombination hotspots, and therefore LD-based analysis provides evidence consistent with the known functional role of the 7-mer motif in stimulating meiotic recombination hotspots. Furthermore, SNPs in the positive set are more enriched (25 out of 48) with significant associations (LDsplit p-value < 0.05) with proximal hotspots than the negative set (11 out of 22).Therefore, LDsplit supports the association between the 7-mer motif and proximal hotspots.

**Figure 4:**
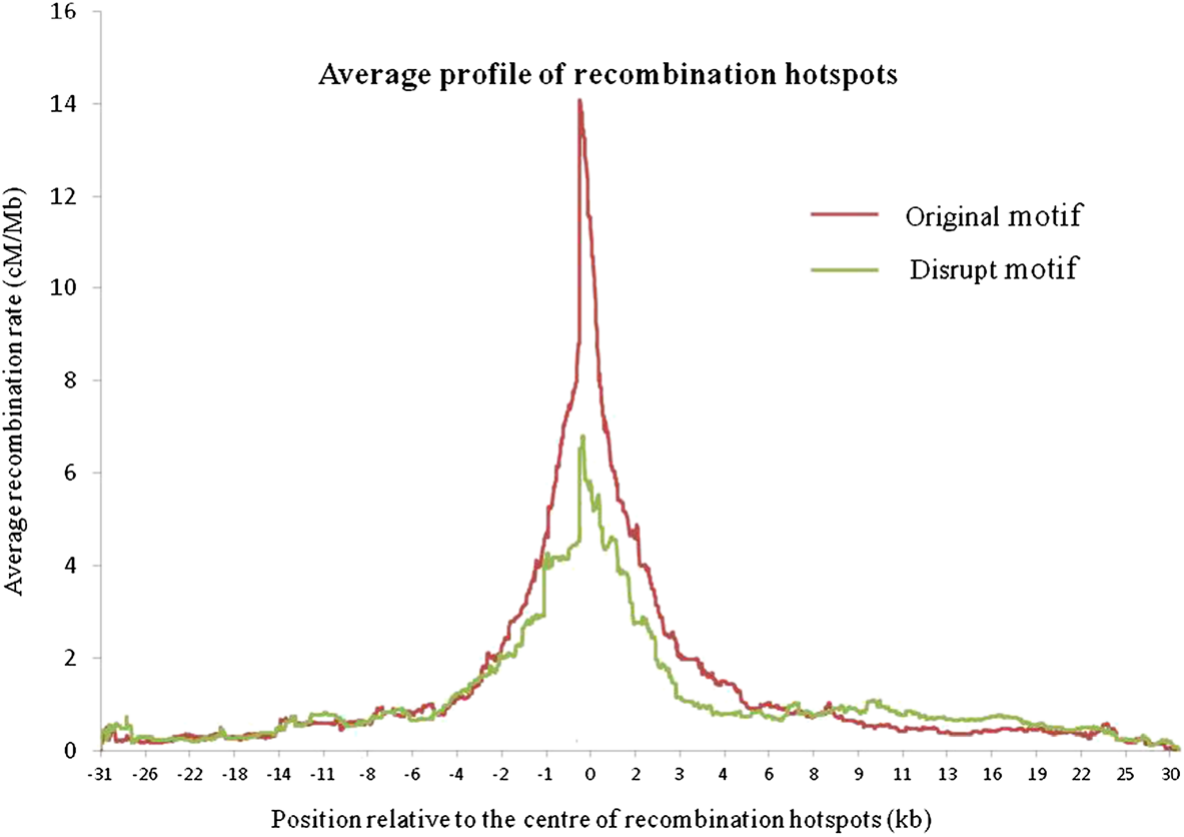
**Comparison of average strengths of hotspots between SNP alleles that preserve and disrupt the PRDM9 motif of CCTCCCT.** We collected hotspot-SNP pairs with significant LDsplit p-values where the SNPs are inside PRDM9 motif. For SNP alleles preserving the motif form of CCTCCCT, the allele-specific recombination profiles of the hotspots are averaged into the red profile in the figure. For the same list of hotspot-SNP pairs, the blue profile plots the average recombination hotspots estimated from haplotypes of SNP alleles that disrupt the CCTCCCT motif.

To further investigate the disrupting effect of SNPs inside the 7-mer motif on the intensities of proximal hotspots using LD patterns, we compared recombination profiles between two SNP alleles as follows. For each of the 77 SNPs, we cut a window of haplotypes flanking the SNP as input to LDsplit, which will calculate the two recombination profiles from the subsets of haplotypes corresponding to two alleles of the SNP. One allele preserves the original form of the 7-mer motif of CCTCCCT, while the other allele disrupts the motif. Then, we aligned the 77 pairs of recombination profiles at the centers of the 15 proximal hotspots, and calculated the average recombination profiles of the two categories (*i.e.* from alleles that preserve the original motif and alleles that disrupt the motif). As shown in Figure 4, the disrupting allele indeed has a lower average intensity than the original allele, thereby confirming that the 7-mer motif stimulates meiotic recombination hotspots. In [4], Myers and colleagues also plotted a comparison of recombination profiles between two motif forms, but their comparison was between different motif forms and different hotspots along the reference human genome. In contrast, we compared the recombination profiles between two alleles of the same SNP and the same hotspot.

### Motif finding guided by LDsplit

In the previous section, we have shown that LDsplit confirms the 7-mer motif as a *cis*-regulator element for meiotic recombination hotspots. However, our goal is to use LDsplit to select SNPs whose positions can guide the discovery of DNA sequence motifs. Figure 3 shows that some SNPs inside the 7-mer motif may not have significant LDsplit p-values, whereas some SNPs outside the motif can have significant p-values. In this section, we will demonstrate that, despite false positives and false negatives, LDsplit is able to provide signals that are sufficient for the detection of the 7-mer motif, by narrowing down to the target genomic regions.

From each of the aforementioned 70 SNPs inside the 7-mer motif, we extended to a window of 101 SNPs (with 50 SNPs on each side). Then we ran LDsplit on the haplotypes of the SNP window to get p-values of SNPs in the window. From the 70 windows, totally 332 SNPs were collected, each of which had a significant LDsplit p-value (p < 0.05). Given reference DNA sequences (human genome assembly Build 36.1, hg18, March 2006, UCSC genome browser) and SNP physical locations in the reference genome, each of the 332 candidate SNPs was extended to a window of 101 DNA bases (50 bases on each side), by cutting the reference sequences flanking the candidate SNPs. Then, we ran the MEME program [20] (version 4.8.1, release date Feb. 7, 2012) on the 332 DNA sequences, setting appropriate parameters for MEME *e.g.* ‘one motif occurrence per sequence’ (OOPS), minimum sites 10 and motif length between 10 to 20, *etc.* (see Section C “Motifs flanking randomly selected SNPs found by MEME” in Additional file 2: Supplementary materials). For comparison, we also randomly selected SNPs (*i.e.* regardless whether their LDsplit p-values are significant), and extended them into 101-base DNA sequences as input for MEME, which was run with the same parameters. To get stable results of comparison, we carried out such random tests for three groups Additional file 3. Additional file 3 contains DNA sequences flanking the SNPs found by LDsplit to be significantly associated with hotspots, and DNA sequences flanking randomly selected SNPs.

**Figure 5:**
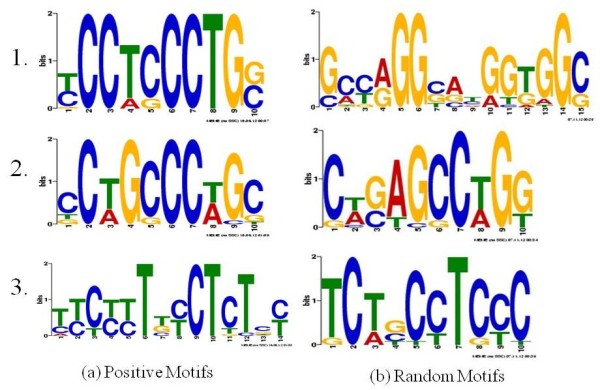
**Motifs reported by MEME from the DNA sequences. (a)** On left side, motifs are from DNA sequences flanking SNPs with significant LDsplit p-values in the windows containing PRDM9 binding motif of CCTCCCT. **(b)** On the right side are motifs from DNA sequences flanking SNPs randomly selected from the same windows.

In Figure 5, motifs found by MEME from the above DNA sequences are ranked by their significance scores (*e.g.* motifs in the first row have the smallest E-value by MEME). The motifs on the left (Figure 5(a)) are from SNPs with significant associations with proximal hotspots as selected by LDsplit, and are called *positive* motifs. From this list, we can see that the top motif in Figure 5(a) matches the known 7-mer motif CCTCCCT very closely. The second significant motif in Figure 5(a) also closely matches the 7-mer motif, except for an insertion of base G after the third position. By contrast, Figure 5(b) shows a group of top random motifs from one of the three groups of SNPs randomly selected without the guide of LDsplit. Clearly, none of the random motifs matches the 7-mer motif closely. Therefore, using the 7-mer motif as a benchmark case study, we have shown that LDsplit can narrow down to target genomic regions, as a guide for motif-finding algorithms like MEME to discover *cis*-regulatory DNA motifs stimulating meiotic recombination hotspots.

In our method, the input to MEME consists of short DNA sequences (101 bases long) flanking SNPs of significant association with hotspots, as detected by LDsplit. By contrast, most existing methods for finding motifs of recombination hotspots use DNA sequences under whole hotspots (2 kb – 10 kb long) as input [3,4]. In the following, we compare the two types of methods and show that, by focusing on short DNA sequences around SNPs, LDsplit can improve accuracy of motif finding. We first selected two groups of hotspots from the same genome-wide list of hotspots as previously used (i.e. estimated by LDhat from HapMap phase 2 data, European population). The first group consists of 15 hotspots that are the same as we used LDsplit and MEME to detect CCTCCCT, the core 7-mer motif of PRDM9. The other group of 15 hotspots was selected randomly, which may or may not contain the 7-mer motif. The locations and lengths of the hotspots are listed in Additional file 1: Tables S1 and S2 in Supplementary materials. Then, for each of the two groups, the DNA sequences under the hotspots were cut and fed into MEME, using the same parameters as we did for LDsplit based motif finding (Figure 5). For each of the two groups, the top 10 motifs ranked by significance (E-values) were recorded (see Additional file 1: Figures S5 and S6 in Supplementary materials). On the list of motifs from the first group of hotspots (Additional file 1: Figure S5), the second motif contains the 6-mer “CCTCCC” as sub-motif, but none of the other motifs is similar with the 7-mer motif. Likewise, from the second group of hotspots, only the third motif and fifth motif contain “CCTCCC” as sub-motif (Additional file 1: Figure S6). Therefore, for the 7-mer core motif of PRDM9, the accuracy of motif finding using the whole sequences under hotspots is not as good as the motif finding guided by SNPs selected by LDsplit. One reason could be that DNA sequences of whole hotspots are much longer than those around SNPs, and hence contain various enriched patterns besides the true motifs, which tend to weaken the true signals. Nevertheless, the other enriched motifs might also have regulatory functions for recombination hotspots, and need further study.

In the above, we have demonstrated the usefulness of LDsplit for the detection of recombination associated motifs, using the 7-mer core motif of PRDM9 as an example. Several recent papers suggest that PRDM9 may be responsible for more recombination hotspots in mammalian genomes than previously appreciated [13,9,22]. However, finding and analysis of DNA motifs responsible for meiotic recombination hotspots are still challenging in many situations. Even for species in which PRDM9 is known to determine recombination hotspots, the specific DNA motifs may not be clear, due to various reasons such as rapid evolution and extensive diversity of PRDM9 zinc finger arrays, variation of genetic and epigenetic contexts flanking the binding sites of PRDM9. In [13], for example, although a consensus motif was found to be present in at least 73% of mouse hotspots, the consensus motif is not the same as the putative PRDM9 binding site generated by ZIFIBI database [23] from zinc fingers in the PRDM9 protein. Moreover, for human and mouse, some transcription factor binding sites in addition to the PRDM9 motif may also regulate recombination hotspots [24,10]. For other species, PRDM9 is either known to be not functional for hotspots (*e.g.* dogs [25]) or a protein with similar functions as PRDM9 has not been found. Sequence based detection of DNA motifs would still be important for these species. Therefore, for species with or without PRDM9 determining initiation of recombination hotspots, LDsplit will be useful for understanding DNA motifs associated with meiotic recombination. In addition to guiding motif finding, LDsplit can also facilitate detailed visualization and analysis of individual hotspots with specific functions, *e.g.* immunology related hotspots in the major histocompatibility complex (MHC) region.

### Finding simulated motifs

To evaluate the performance of LDsplit, we conducted a simulation study using a Python program based on simuPOP [21], which we developed earlier [18] to simulate the evolutionary process of a recombination hotspot. A causal SNP is inserted as a critical component of the simulation. In each event of meiosis in the simulated evolution, the probability of crossover is determined by the allele of the causal SNP (see Implementation for details). Running on simulated SNP data, LDsplit detected association of a hotspot with SNPs with MAF ≥ 0.3 (including the causal SNP) and calculated their p-values, in the same way as it does to real HapMap SNP data. Table 1 lists values of key parameters in our simulation experiment. We simulated five populations, and from each population we randomly sampled 10 subsets, each consisting of 90 individuals (*i.e.* 180 haplotypes). The performance of LDsplit on the 50 datasets was consistent with our previous simulation tests [18] (data not shown).

**Table 1:** Parameter setting on simulated data

**Causal SNP position (kb)**	**Window length (kb)**	**Hotspot center (kb)**	**Hotspot width (kb)**	**Beginning hot allele frequency (%)**	**Ending hot allele frequency (%)**	**# sample subset**	**Sample subset size**
100	200	100	2	99	50	10	180

**Figure 6:**
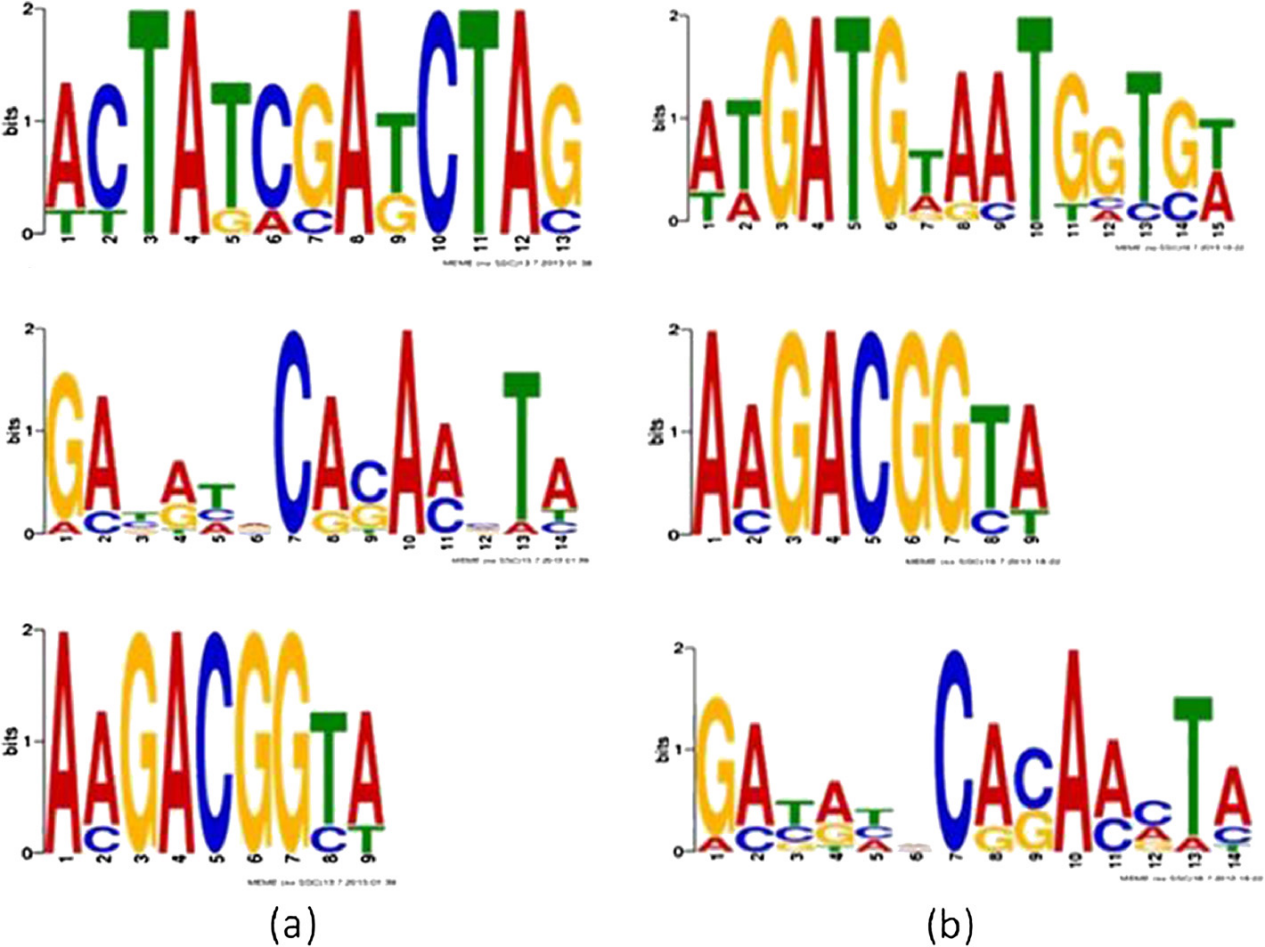
**Simulation tests of motif finding by LDsplit and MEME.** Motifs found by LDsplit and MEME in the simulated DNA sequences containing inserted motifs: **(a)** Inserted motif of “ACTATCGATCTAG” found as top hit by MEME; **(b)** inserted motif of “ATGATGTAATGGT” found by MEME.

Next, we tested whether the screening of SNPs by LDsplit can help find causal sequence motifs in the simulated data. From the 50 simulated datasets above, we collected totally 60 candidate SNPs with significant LDsplit p-values (p < 0.05), each of which was extended to a window of 101 DNA bases (50 bases on each side). Among the 60 DNA sequences, we inserted two randomly generated causal motifs “ACTATCGATCTAG” and “ATGATGTAATGGT” in locations where they span the causal SNPs, thereby modelling the causal effect of the motifs in stimulating meiotic recombination. In case the causal SNP is not included in the 60 SNPs due to false negative of LDsplit, the motif is not inserted. Then we ran MEME on the 60 DNA sequences to identify motifs, with the same setting of parameters of MEME as for finding the 7-mer motif of CCTCCCT. As shown in Figure 6(a) and Figure 6(b), the motifs candidates were ranked by statistical significance, and the top motif candidates found by MEME match the causal motifs “ACTATCGATCTAG” and “ATGATGTAATGGT” very closely. This simulation demonstrates that LDsplit is able to screen for genomic loci that are more likely to contain hotspot associated DNA sequence motifs.

### Effect of biased gene conversion

While the idea of LDsplit appears simple, there are subtle caveats because a meiotic recombination hotspot tends to kill itself through biased gene conversion (BGC) [26,27]. Hellenthal *et al.*[28] suggested that, due to BGC, it is likely that an extant hot allele may experience no more crossovers than the cold allele in the history of a population, and therefore it is difficult to detect the allele-specific effect of DNA sequence polymorphisms on the intensity of a recombination hotspot from LD patterns. This conclusion was drawn from a mathematical model to estimate the probability that a chromosome with the hot (and cold) allele of a SNP experienced a crossover in the previous generation, taking into account of BGC. However, their mathematical model also predicted that, when the probability of crossover of a hot allele is say 20 times higher than the cold allele, the number of crossover events in the sub-population of the hot allele is about 3.4 times that of the cold allele in the transmission from the previous generation. This is the case for the DNA2 hotspot and FG11 SNP, as reported by sperm typing [16]. Hence, the mathematical model of Hellenthal *et al.* offers an explanation for the success of LDsplit in predicting the association between the DNA2 hotspot and FG11 SNP [18]. Although the general conclusion in [28] highlighted the difficulty of association studies for recombination hotspots using LD patterns, it does not rule out the effectiveness of LD-based methods like LDsplit, especially for those SNPs with a big difference in hotspot penetrance between two alleles. Indeed, LDsplit has been verified on both real and simulated data, with promising performances. In addition to correctly associate the DNA2 hotspot with the FG11 SNP, it identified an 11-mer motif whose complement closely matches the 13-mer binding motif of PRDM9 [18].

In this paper, we confirmed once gain the prediction of the mathematical model of Hellenthal *et al.*[28]. As Figure 3 shows, for 22 out of 70 SNPs inside the 7-mer motif CCTCCCT, the disrupting allele, which is supposed to be cold, has higher intensities in proximal hotspots than the “hot” allele that preserves the original motif form. This is probably due to the effect of BGC. Meanwhile, the fact that in 48 out of 70 cases the hot allele still has higher LD-based recombination rate (Figure 3) suggests that the 7-mer motif has a strong stimulating penetrance on proximal recombination hotspots. Whereas we did not give any theoretical explanation for the effectiveness of LDsplit in spite of BGC in our previous work [18], here we have made the connection between experimental results and mathematical theory of population genetics. Our new experiments and analysis suggest that LDsplit may be more likely to be effective when there is a big difference in hotspot penetrance between two alleles of an associated SNP (*e.g.* the DNA2-FG11 case).

### Future work

Since our presentation of LDsplit software in a recent conference [29], several researchers have inquired, downloaded and used LDsplit, showing their interests in this software tool. For broader applications and impacts on the biological research, we plan to further improve LDsplit and continue Bioinformatics analysis of recombination hotspots as follows. First, as LDsplit is based on the computation intensive algorithm of LDhat, it is still time consuming for large data sets. Thus, we will speed up LDsplit by parallel computing, algorithm optimization, and pre-computing of results on large-scale data (*e.g.* HapMap SNPs). Recently we have obtained significant speedup for LDhat algorithm by optimization and parallel computing [30]. Second, as the biased gene conversion (BGC) plays a key role in the success of LDsplit, we will investigate the relation between recombination hotspots and BGC (see [31] for recent research in this direction).

One implication of the discovery of PRDM9 is that epigenetic factors (*e.g.* DNA methylation and histone modification) play important roles in the regulation of recombination hotspots. Several recent studies have pointed to this connection [10,13,32]. Recently we have made an effort to integrate genetic and epigenetic factors for regulating meiotic recombination into one predictive model, with promising results [12]. An important future work is to incorporate epigenetic factors into the detection of *cis*-regulatory DNA motifs.

## Conclusions

In this paper we presented LDsplit, an open source software tool for predicting *cis*-regulatory motifs of meiotic recombination hotspots through SNP analysis. With its graphical user interface which allows convenient interactive and integrative analysis of hotspots and associated DNA sequences, LDsplit will be a useful tool for many researchers working in areas including (but not limited to) DNA recombination, genomic evolution, disease gene mapping, and genome instability. In this paper, we demonstrated the usefulness and accuracy of LDsplit by testing on the 7-mer DNA motif of CCTCCCT which is bound to by PRDM9 and a well-known *cis*-regulator of recombination hotspots. We showed that SNP alleles disrupting the 7-mer motif have lower hotspot intensities estimated by LDsplit. Moreover, LDsplit is able to guide the discovery of the 7-mer motif and simulated motifs by the MEME software. Such a Bioinformatics approach is promising for the discovery of *cis*- and *trans*-regulators, to uncover the molecular machinery regulating recombination hotspots and genome stability.

## Availability and requirements

**Project name:** LDsplit.

**Project home page: **http://www.ntu.edu.sg/home/zhengjie/software/LDsplit.htm.

**Operating system(s):** Windows, Linux, Mac OS.

**Programming language:** Java.

**Other requirements:** JDK, JFreechart package.

**License:** GNU General Public License.

**Any restrictions to use by non-academics:** None.

## Abbreviations

BGC: Biased gene conversion; MAF: Minor allele frequency; SNP: Single nucleotide polymorphism; LD: Linkage disequilibrium; GUI: Graphical user interface.

## Competing interests

The authors declare that they have no competing interests.

## Authors’ contributions

JZ and TM envisioned the idea behind the LDsplit algorithm. JZ, PY and JG implemented the LDsplit algorithm in a Java software tool and designed the graphical user interface. PY conducted computational experiments running and validating LDsplit, and MW collected data of DNA sequences and motif locations. PY, MW and JZ drafted the manuscript, with input from TM and CKK. All authors read and approved the final manuscript.

## Supplementary Material

Additional file 1 **Supplementary materials.** The file of “Supplementary_materials.pdf” contains several figures and additional experimental results mentioned in this paper, including: (1) LDsplit’s confirmation of the association between FG11 SNP and DNA2 hotspot on chromosome 6 discovered by sperm typing; (2) the interface of LDsplit for loading input data and setting parameters; (3) 5 groups of top DNA sequence motifs found by MEME from DNA sequences flanking randomly selected SNPs. Click here for file

Additional file 2 **SNPs inside 7-mer core motif of PRDM9.** Additional file 2 is an Excel file “70_SNP_motif_info.xlsx” containing the detailed information of the 70 selected SNPs that are located inside the 7-mer motif of CCTCCCT. The columns are chromosome numbers, SNP rs numbers, SNP physical locations, SNP allele that preserves the motif form, SNP alleles that disrupt the motif, DNA strand on which the motif occurs (forward strand 5′ to 3′ marked as “+” and the reverse complementary strand marked as “−” ) and disrupt motif forms (where the disrupting SNP allele is marked in brackets). Click here for file

Additional file 3 **DNA sequences for motif finding.** Additional file 3 is a zip file containing 4 FASTA files and one excel file. The file of “Positive_Group.FASTA” contains 332 DNA sequences each of length 101 bases extended from 332 SNPs with significant associations with proximal hotspots reported by LDsplit from the 70 SNP windows studied in the paper. Each of the rest three FASTA files (i.e. “Negative_Group_1.FASTA”, “Negative_Group_2.FASTA” and “Negative_Group_3.FASTA”) contains 101-base DNA sequences from the three groups of randomly selected SNPs. The excel file “Positive_SNP_info.xlsx” contains information of the 332 SNPs (e.g. chromosome numbers, physical locations, rs numbers, and the flanking 101 DNA sequences). Click here for file
