# Supplementary material for: LDsplit: screening for cis-regulatory motifs stimulating meiotic recombination hotspots by analysis of DNA sequence polymorphisms
Source: BMC Bioinformatics. 2014 Feb 17;15:48. doi: 10.1186/1471-2105-15-48 (PMC3936957; doi:10.1186/1471-2105-15-48)
Supplement: Additional file 1 — Supplementary materials. The file of “Supplementary_materials.pdf” contains several figures and additional experimental results mentioned in this paper, including: (1) LDsplit’s confirmation of the association between FG11 SNP and DNA2 hotspot on chromosome 6 discovered by sperm typing; (2) the interface of LDsplit for loading input data and setting parameters; (3) 5 groups of top DNA sequence motifs found by MEME from DNA sequences flanking randomly selected SNPs. [file 1471-2105-15-48-S1.doc]

**Supplementary Materials**

LDsplit: Screening *cis*-regulatory motifs stimulating meiotic recombination hotspots by analysis of DNA sequence polymorphisms

### Peng Yang^1,2^, Min Wu^1^, Jing Guo^1^, Chee-Keong Kwoh^1^, Teresa M. Przytycka^3^ and Jie Zheng^1,4§^

^1^ Bioinformatics Research Centre (BIRC), School of Computer Engineering, Nanyang Technological University, 50 Nanyang Avenue, Singapore 639798

^2^ Institute for Infocomm Research (I2R), A*STAR (Agency for Science, Technology, and Research), 1 Fusionopolis, Singapore 138632

^3^ NCBI, NLM, National Institutes of Health, 8600 Rockville Pike, Bethesda, Maryland 20894, USA

^4^ Genome Institute of Singapore, A*STAR, Biopolis, Singapore 138672

^§^Corresponding author

1. **Confirming sperm typing case of FG11 SNP and DNA2 hotspot**

In this newer version, LDsplit was implemented in Java language with a user-friendly interface as shown in Figure 2. This greatly facilitates the analysis of data, providing users with an integrative view of genomic context of hotspots (e.g. flanking DNA sequences). We tested the newly implemented LDsplit on HapMap SNP data to predict the association of the FG11 SNP with the DNA2 hotspot, previously reported by sperm typing experiment in our previous work . As shown the Figure S1, the T allele of the FG11 SNP, which corresponds to increased recombination rate in the sperm typing result, has a higher recombination rate than the cold C allele. Moreover, in all 3 populations, FG11 has significant association with the DNA2 hotspot (p-values in African, Asian, and European populations are 0.02822, 0.0411 and 0.00956 respectively, all less than the p-value threshold of 0.05). This result demonstrates the efficacy of LDsplit to correctly identify SNP-hotspot pairs previously only observed in sperm typing experiments. Note that, while sperm typing is limited to a few short regions due to high cost and technical limitations, the computational method of LDsplit can be applied genome-wide to thousands of hotspots in human as well as other species.

**(a) African population (Yoruba in Ibadan, (b) Asian population (Chinese and**

**Nigeria, or YRI) Japanese, or CHB+JPT)**

**(c) European population (Utah Residents with**

**Northern and Western European Ancestry, or CEU)**

**Figure S1.** The LDsplit results for FG11-DNA case to confirm sperm typing results

1. **Interface of LDsplit for input data and parameters**


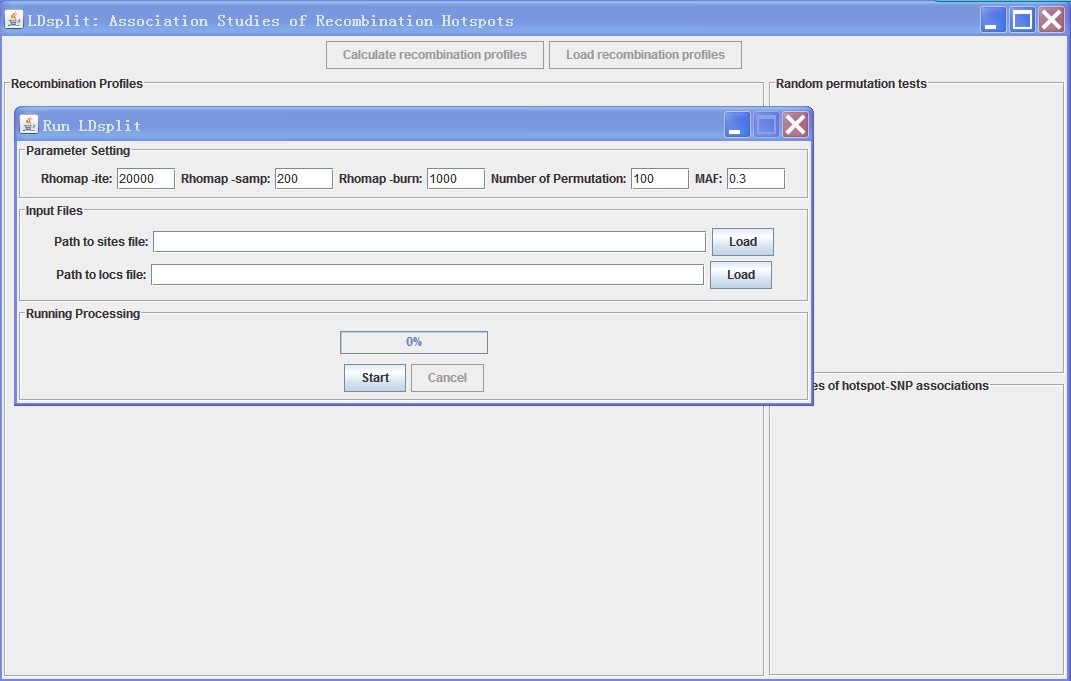


## Figure S2. The panel for importing input data and setting parameters to run LDsplit

1. **Motifs flanking randomly selected SNPs found by MEME**


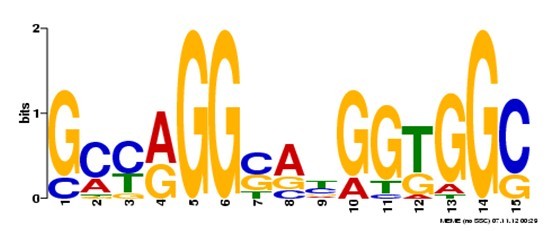

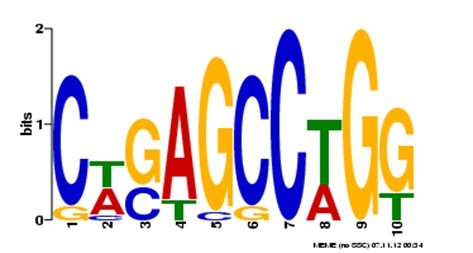


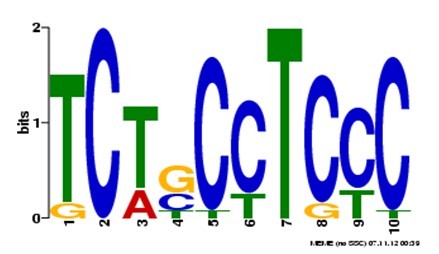


**Figure S3(a).** Motifs found in negative set 1


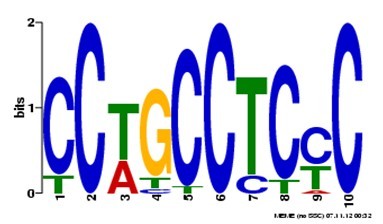

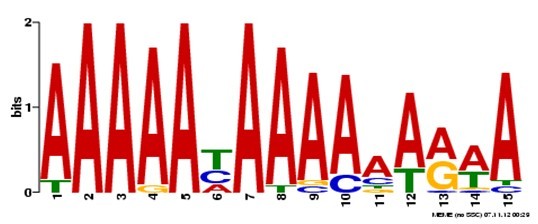

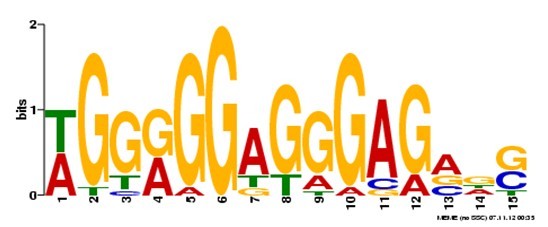


**Figure S3(b).** Motifs found in negative set 2


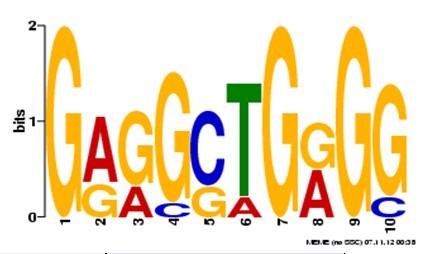

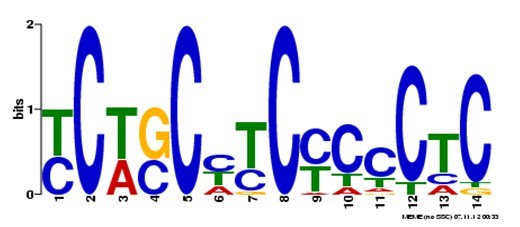

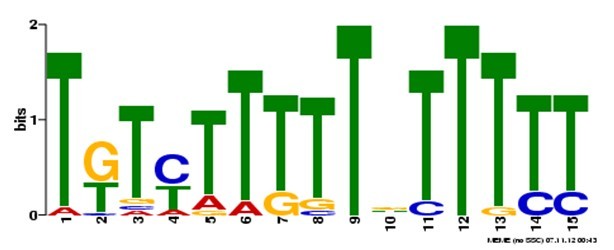


**Figure S3(c)**. Motifs found in negative set 3

The parameters setting of the MEME experiments in this paper are as following:

Optimum number of sites for each motif within the limits:

Minimum: 10 Maximum: 20

Optimum width of each motif within the limits:

Minimum: 10 Maximum: 15

Other parameter setting are default values in the MEME website.

1. **Correlation analysis for allelic variation in human PRDM9**

Recent studies in human , mouse and chimp suggest an important role of PRDM9 in determining recombination rates. To explore the likelihood that the observed recombination hotspot variation is simply due to the allelic diversity of PRDM9, we carried out hotspot-SNP association study for SNPs inside the coding region of the PRDM9 protein. In HapMap phase 2 dataset, there are three SNPs within the Prdm9 gene, namely rs2914281, rs1874165 and rs6874441. However, running on the HapMap Phase 2 data (CEU population), LDsplit was unable to estimate the association of these SNPs with proximal hotspots as their MAFs are too small (0.017, 0.058 and 0.042 respectively).

Nonetheless, the association of the PRDM9 SNPs with hotspots can still be measured by their correlations with other SNPs that are significantly associated with hotspots as found by LDsplit, assuming that a SNP with allele distribution highly correlated with causal SNPs is also associated with hotspots. To achieve this,
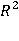
 (*R squared*), correlation coefficient of the frequencies between SNPs, is calculated for a pair of SNPs. Given a sample of haplotypes, defined as *H*, and two SNPs consisting of alleles A/a and B/b respectively, the
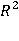
 correlation coefficient between the two SNPs is defined as:


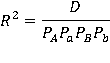


where
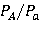
 are frequencies of alleles A/a in the population (similarly for
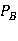
 and
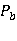
) and
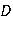
 is degree of Linkage Disequilibrium existing between two loci defined as:


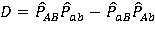


where
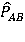
is the observed frequency of the allele combination *AB* in the haplotype data (similarly for
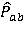
,
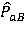
 and
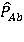
).

Hotspot-SNP associations for PRDM9 are evaluated as follows. We collected totally 1673 SNPs, each having significant LDsplit p-value (p < 0.05) in association with proximal hotspots in Chromosome 5. For each of the 3 SNPs from PRDM9, we calculate its
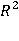
 correlation coefficient with each of the 1673 significant SNPs.

The histograms in Figure S4 (a)-(c) show the distribution of the correlation values spanning from −5 to +5. We observed that the correlations are approximately in normal distribution with mean at zero, indicating that allele diversity in PRDM9 has low correlation with recombination hotspot variation in Chromosome 5. It is interesting that there are small enrichments at −1.5 and +2.0 in correlations for SNP rs1874165 as shown in Figure S4(a), which can be investigated in future although beyond the scope of this paper.

In summary, our study suggests that allelic diversity in human PRDM9 is unlikely to be the dominant driving force in shaping the detailed variation of recombination hotspots. Since PRDM9 is a prominent *trans*-regulator of recombination hotspots, its own allelic diversity could impact the global landscape of recombination hotspots. However, our paper is focused on *cis*-regulatory elements of hotspots, aiming to identify regulatory forces that complement (rather than replace) the impact of *trans*-factors like PRDM9.

| 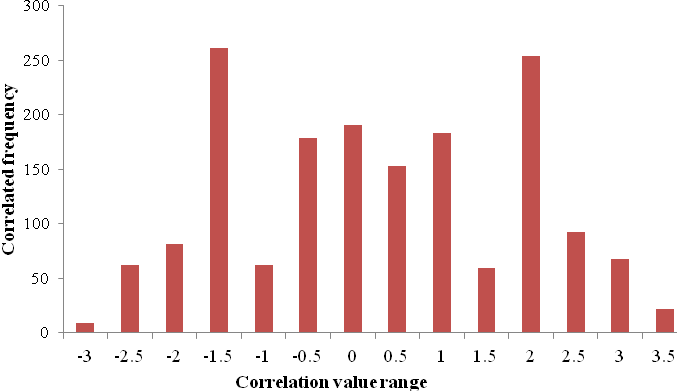   1. SNP rs2914281 | 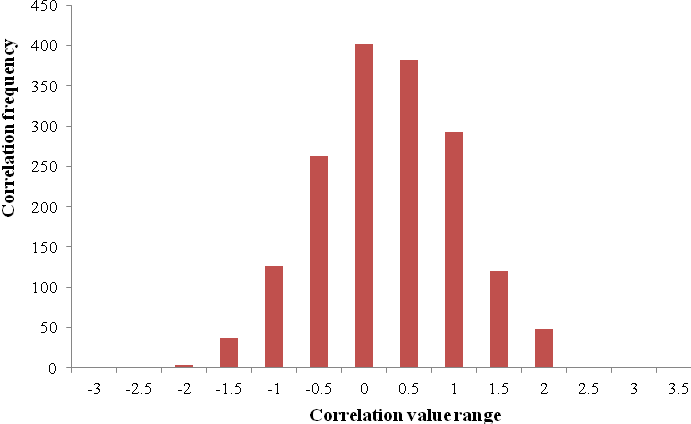   1. SNP rs1874165 |
| --- | --- |
| 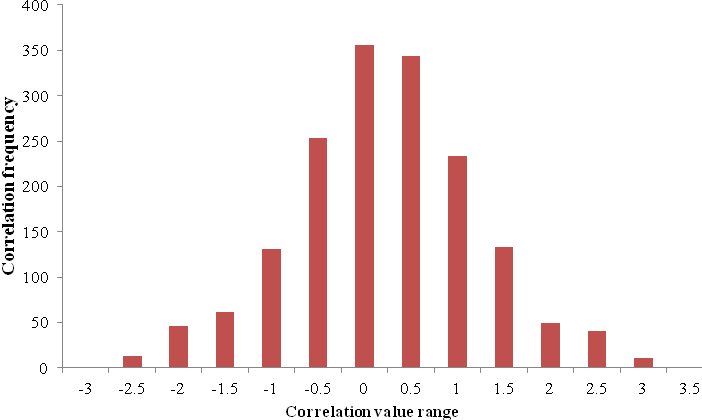   1. SNP rs6874441 | |

## Figure S4. Distributions of correlations of three SNPs inside PRDM9 coding regions with significant SNPs found by LDsplit on Chromosome 6

1. **Motif finding using DNA sequences of whole hotspots**

**Table S1.** Recombination hotspots with motif PRDM9 inside (group 1)

| Chromosome No. | Start Position (bp) | End Position (bp) | Length of hotspots (bp) |
| --- | --- | --- | --- |
| 1 | 112512001 | 112515001 | 3,000 |
| 3 | 37577001 | 37580001 | 3,000 |
| 6 | 32979001 | 32982001 | 3,000 |
| 8 | 119417001 | 119421001 | 4,000 |
| 8 | 53092001 | 53095001 | 3,000 |
| 9 | 92656001 | 92661001 | 5,000 |
| 11 | 115070001 | 115075001 | 5,000 |
| 11 | 4612001 | 4617001 | 5,000 |
| 13 | 53403000 | 53406000 | 3,000 |
| 14 | 82329000 | 82332000 | 3,000 |
| 14 | 67060000 | 67065000 | 5,000 |
| 15 | 93890000 | 93894000 | 4,000 |
| 15 | 72374000 | 72379000 | 5,000 |
| 15 | 67835000 | 67839000 | 4,000 |
| 16 | 6153001 | 6156001 | 3,000 |

**Table S2.** Recombination hotspots which may not contain PRDM9 motif (group 2)

| Chromosome No. | Start Position (bp) | End Position (bp) | Length of hotspots (bp) |
| --- | --- | --- | --- |
| 21 | 17204000 | 17209000 | 5,000 |
| 19 | 52542001 | 52545001 | 3,000 |
| 19 | 51261001 | 51265001 | 4,000 |
| 18 | 20695001 | 20698001 | 3,000 |
| 15 | 58920000 | 58924000 | 4,000 |
| 16 | 20459001 | 20464001 | 5,000 |
| 12 | 32805001 | 32810001 | 5,000 |
| 11 | 90605001 | 90609001 | 4,000 |
| 10 | 76197001 | 76201001 | 3,000 |
| 8 | 121337001 | 121340001 | 3,000 |
| 7 | 139224001 | 139227001 | 3,000 |
| 6 | 140017001 | 140020001 | 3,000 |
| 5 | 136668001 | 136673001 | 5,000 |
| 3 | 23716001 | 23721001 | 5,000 |
| 1 | 2064001 | 2068001 | 4,000 |

**Table S3.** Experimental Setting in MEME

| Distribution of motif occurrences | Any number of repetitions |
| --- | --- |
| Number of different motifs | 10 |
| Minimum number of sites | 5 |
| Maximum number of sites | 30 |
| Minimum motif width | 7 |
| Maximum motif width | 15 |


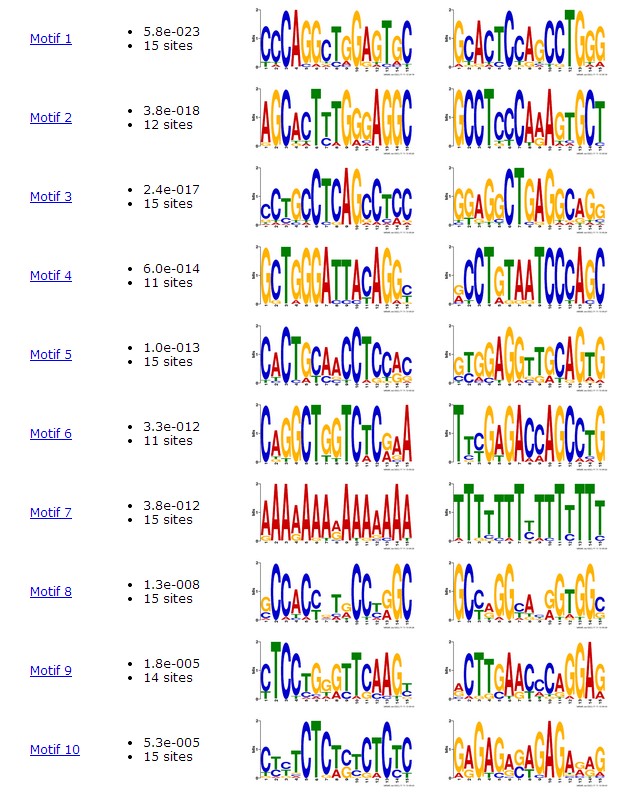


**Figure S5**. Top 10 motifs found by MEME from hotspot sequences of group 1


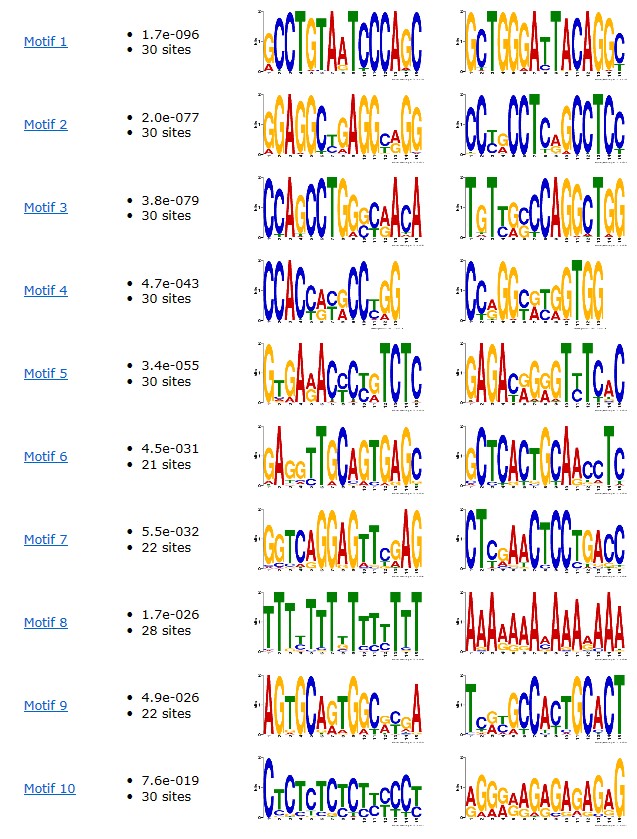


**Figure S6**. Top 10 motifs found by MEME from hotspot sequences of group 2

**References**

1. Jeffreys, A.J. and R. Neumann, *Reciprocal crossover asymmetry and meiotic drive in a human recombination hot spot.* Nature Genetics, 2002. **31**(3): p. 267-71.

2. Zheng, J., et al., *Detecting sequence polymorphisms associated with meiotic recombination hotspots in the human genome.* Genome Biology, 2010. **11**(10): p. R103.

3. Myers, S., et al., *Drive against hotspot motifs in primates implicates the PRDM9 gene in meiotic recombination.* Science, 2010. **327**(5967): p. 876-9.

4. Smagulova, F., et al., *Genome-wide analysis reveals novel molecular features of mouse recombination hotspots.* Nature, 2011. **472**(7343): p. 375-8.

5. Auton, A., et al., *A fine-scale chimpanzee genetic map from population sequencing.* Science, 2012. **336**(6078): p. 193-8.
